# Online Estimation of Allan Variance Coefficients Based on a Neural-Extended Kalman Filter

**DOI:** 10.3390/s150202496

**Published:** 2015-01-23

**Authors:** Zhiyong Miao, Feng Shen, Dingjie Xu, Kunpeng He, Chunmiao Tian

**Affiliations:** 1 Department of Automation, Harbin Engineering University, Harbin 150000, China; E-Mails: miaozhiyong@hrbeu.edu.cn (Z.Y.M.); hekunpeng@hrbeu.edu.cn (K.P.H.); 2 School of Electrical Engineering and Automation, Harbin Institute of Technology, Harbin 150000, China; E-Mail: xdj@hrbeu.edu.cn; 3 Department of Information and Communication Engineering, Harbin Engineering University, Harbin 150000, China; E-Mail: tianchunmiao@hrbeu.edu.cn

**Keywords:** Allan variance, stochastic errors, online estimation methods, nonlinear state-space model, neural-extended Kalman filter

## Abstract

As a noise analysis method for inertial sensors, the traditional Allan variance method requires the storage of a large amount of data and manual analysis for an Allan variance graph. Although the existing online estimation methods avoid the storage of data and the painful procedure of drawing slope lines for estimation, they require complex transformations and even cause errors during the modeling of dynamic Allan variance. To solve these problems, first, a new state-space model that directly models the stochastic errors to obtain a nonlinear state-space model was established for inertial sensors. Then, a neural-extended Kalman filter algorithm was used to estimate the Allan variance coefficients. The real noises of an ADIS16405 IMU and fiber optic gyro-sensors were analyzed by the proposed method and traditional methods. The experimental results show that the proposed method is more suitable to estimate the Allan variance coefficients than the traditional methods. Moreover, the proposed method effectively avoids the storage of data and can be easily implemented using an online processor.

## 1. Introduction

Inertial sensors are valuable sensors for navigation of aircraft systems, vehicles and strategic weapons [1]. However, stochastic errors, inherently present in inertial sensor outputs, significantly affect the performance of inertial sensors. To eliminate the effect of stochastic errors, they should be modeled and identified to properly compensate or filter them before integrating into the navigation system [2–4]. In general, the noise analysis methods used for inertial sensors include online and offline estimation methods. In the offline methods, frequency and time-domain approaches have been used to model the stochastic errors of inertial sensors. As a frequency-domain method, power spectral density (PSD) is commonly used to investigate the stochastic errors of inertial sensors. Although, the PSD-based method is straightforward to estimate the transfer functions of stochastic errors, it is difficult for non-system analysts to understand [5–7]. As a time-domain analysis technique, the Allan variance is a simple and useful method in determining the characteristics of the underlying random processes causing the data noise. It has been widely used for identifying stochastic processes such as quantization noise, white noise, correlated noise, sinusoidal noise, random walk, and flicker noise in inertial sensors [8–10]. Recently, modified Allan variance methods such as sliding average Allan variance [11] and fully and not fully overlapping Allan variance [12] have been developed. However, these methods are time-consuming, offline, and error-prone [13].

Compared to offline methods, online methods have rarely been studied. One online method is reported in [14,15], and another is reported in [16,17]. In [14,15], an equivalent ARMA was used to model the MEMS IMU stochastic errors to obtain a linear Gaussian state-space model, and then a recursive EM algorithm proposed by Elliot and Krishnamurthy [18] was used. The method proposed in [14,15] does not require the storage of any data and can be implemented using an online processor, however, it is only valid for the stochastic errors driven by white noise. Therefore, the quantization noise cannot be estimated directly [19]. Moreover, transformations during the modeling are complex, particularly for the estimation of four parameters. The main advantage of the method proposed in [16,17] is that it models the dynamic expression of Allan variance using exponentially weighted moving average algorithm to solve the complex transformations of a linear state-space model reported in [15]. The method also can estimate any stochastic errors present in raw data. However, it still has two major drawbacks: (I) The additional error can be introduced in the measurement equation by the exponentially weighted moving average algorithm; and (II) the Sage-Husa adaptive Kalman filter used to estimate Allan variance coefficients may cause filter divergence while estimating process noise covariance matrix *Q_k_* and measurement noise covariance matrix *R_k_* simultaneously [20]. Finally, it must be pointed out that the method reported in [16,17] is out of the scope of this paper, because this study focuses on static gyros rather than onboard gyros.

To reduce the complexity of modeling and estimate all five Allan variance coefficients in real time, a new online estimation method different from the above methods is proposed in this paper. In the proposed method, the recursive expressions of Allan variance were modeled directly instead of using exponentially weighted moving average algorithm to obtain an accurate nonlinear state-space model and implemented by a neural-extended Kalman filter (NEKF) algorithm to estimate the Allan variance coefficients. Because the state equation is directly modeled by random errors and zero-mean Gauss white noise, the measurement equation is a recursive expression of Allan variance, and a NEKF algorithm is used to estimate the Allan variance coefficients. The proposed method avoids the limitations of the existing online methods. The experimental results show that the proposed method has a simple modeling and can estimate five Allan variance coefficients online.

This paper is organized as follows: In Section 2, the sources of the stochastic errors of inertial sensors are described. Section 3 reviews the existing online estimation methods based on a linear state-space model. The proposed method comprising the new nonlinear state-space model of Allan variance coefficients and NEKF is described in detail in Section 4. Section 5 discusses the experimental results in two subsections. In the first subsection, an ADIS16405 MEMS sensor was used as the stochastic error source to evaluate the performance of the Allan variance method, existing online estimation methods, and proposed method. In the second subsection, another experiment was conducted to estimate Fiber Optic Gyro (FOG) stochastic errors to verify that the proposed method can estimate the coefficients of five basic stochastic errors simultaneously without any limitation. Finally, the conclusion and future work are summarized in Section 6.

## 2. Stochastic Error Sources in Inertial Sensors

**Figure 1.**
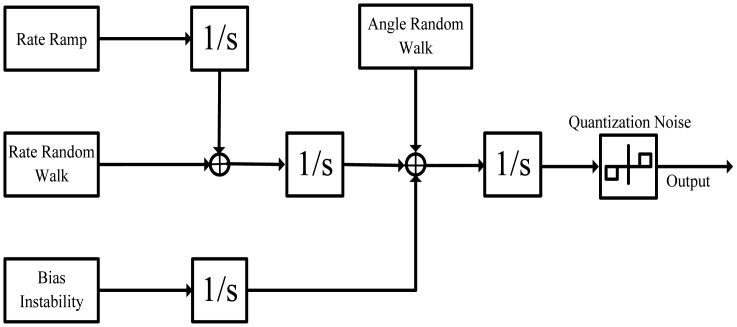
Stochastic model of inertial sensor.

The aim of this section is to discuss the five basic noise terms and give their corresponding differential equations. The stochastic model of inertial sensors is shown in Figure 1. The five basic noise terms in Figure 1 are quantization noise, angle random walk (ARW), bias instability, rate random walk (RRW), and rate ramp. The definitions and their detailed derivations are given in [21].

Quantization noise: This is one of the errors introduced into an analog signal by encoding it in a digital form. It represents the minimum resolution level of the sensor. The Allan variance of quantization noise can be expressed as follows:

(1) $$\delta_{Q}^{2}(\tau )=\frac{3Q^{2}}{\tau^{2}}$$

where *Q* is the quantization noise coefficient, and τ is the sample interval.

ARW: This is a high-frequency noise and characterized by a white-noise rational spectrum on gyro rate output voltages. The differential equation and Allan variance of ARW can be expressed as follows:

(2) $$y_{\text{arw}}=Nv_{1}(t)$$

(3) $$\delta_{\text{ARW}}^{2}(\tau )=\frac{N^{2}}{\tau }$$

where *N* is the ARW coefficient, and *v*_1_(*t*) is the unit white noise.

Bias instability: This noise is originated from the electronics or other components that are susceptible to random flickering. It is also known as flicker noise and approximated by the first-order Gauss-Markov process as follows:

(4) $$y_{f}=\frac{\beta Βv_{2}(t)}{D+B}$$

where *D* is the differential operator, β is the reciprocal correlation time and can be determined by autocorrelation, *B* is the flicker noise parameter, and *v*_2_(*t*) is the unit white noise.

The Allan variance of bias instability can be expressed as follows:

(5) $$\delta_{f}^{2}(\tau )={\left(\frac{B}{0.6648}\right)}^{2}$$

RRW: This is a random process of uncertain origin, possibly a limiting case of an exponentially correlated noise with a very long correlation time. It is associated with the PSD rate. The differential equation and Allan variance of RRW can be expressed as follows:

(6) $$y_{\text{rrw}}=\frac{Kv_{3}(t)}{D}$$

(7) $$\delta_{\text{rrw}}^{2}(\tau )=\frac{K^{2}\tau }{3}$$

where *K* is the RRW coefficient, and *v*_3_(*t*) is the unit white noise.

Rate ramp: The error terms considered so far have random character. This is also probably because of a very small acceleration of the platform in the same direction and persisting over a long period of time. Because this noise has nonrational spectra, the second-order Gauss-Markov process is used as the approximation:

(8) $$y_{rr}=\frac{Rv_{4}(t)}{D^{2}+\sqrt{2}w_{0}D+{w_{0}}^{2}}$$

where *R* is the ramp noise parameter, ω_0_ is the undamped natural frequency of this second-order system and needs to be determined, and *v*_4_(*t*) is the unit white noise.

The Allan variance of ramp noise can be expressed as follows:

(9) $$\delta_{rr}^{2}=\frac{R^{2}\tau^{2}}{2}$$

The Allan variance is a method of representing root-mean-square (RMS) random drift error as a function of averaging times [22]. As shown in [5], the above stochastic errors can be identified from the standard Allan variance plot. The Allan variance coefficients and the slope of the corresponding curves are shown in Table 1.

## 3. Online Estimation Methods Based on Linear State-Space Model: A Review

An online estimation method based on a linear state-space model was first introduced by Ford and Evans in [14]. Only two random errors, ARW and RRW, were estimated using this method. Based on this method, another online estimation method was developed to estimate three random errors, ARW, RRW, and bias instability, by Saini and Rana [15]. Although there are differences between these methods, in some sense, the method proposed in [15] is an extended version of that proposed in [14]. For convenience, the method proposed in [15] was used as the existing method in the rest of this paper. In this paper, the proposed online estimation method was compared to the existing method. To compare the complexity of the modeling process and select between the existing and proposed methods in this paper, the existing method was reviewed first. The existing method mainly contains two steps: The first step is to model the stochastic errors to obtain a linear state-space model, and the second step is to use the finite-dimensional filters to estimate the coefficients of stochastic errors. Herein, the two steps are introduced in Subsections 3.1 and 3.2. The detailed derivations of this method are reported in [15].

### 3.1. Linear State-Space Model

The main steps of modeling the stochastic errors in a discrete linear state-space model can be described as follows:

Step 1 Model an equivalent ARMA model [23] driven with a single white noise as follows:
(10) $$Y(t)=y_{\text{rrw}}(t)+y_{f}(t)+y_{rr}(t)$$
  where *Y*(*t*) is used to represent the mixture of *y_rrw_*(*t*), *y_f_*(*t*), and *y_rr_*(*t*).
Step 2 Substituting Equations (4), (6) and (8) into Equation (10), we obtain
(11) $$\begin{array}{ll} D(D+\beta )(D^{2}+\sqrt{2}w_{0}D+{w_{0}}^{2})Y(t) & =D(D+\beta )(D^{2}+\sqrt{2}w_{0}D+{w_{0}}^{2})Kv_{2}(t)+\\ & D(D^{2}+\sqrt{2}w_{0}D+{w_{0}}^{2})\beta Bv_{3}(t)+D(D+\beta )Rv_{4}(t) \end{array}$$
  The left hand side of Equation (11) is an equivalent AR model, and the right hand side of Equation (11) is an equivalent MA model. Moreover, the Equation (11) can be rewritten as:
(12) $$\ddddot{Y}(t)+c_{1}\dddot{Y}(t)+c_{2}\ddot{Y}(t)+c_{3}\dot{Y}(t)+c_{4}Y(t)=\theta_{0}\dddot{r}+\theta_{1}\ddot{r}+\theta_{2}\dot{r}+\theta_{3}r$$
  where the superscript of *Y*(*t*) and *r* represent the differential of *Y*(*t*) and *r*. *c*_1_, *c*_2_, *c*_3_, *c*_4_ and θ_0_, θ_1_, θ_2_, θ_3_ are coefficients of equivalent ARMA model, the *r* is the white noise.
  Referring to [15] to solve Equation (12), here we only give the results as follows:
(13) $$\begin{array}{l} c_{1}=\beta +\sqrt{2}r\\ c_{2}=\sqrt{2}r\beta +r^{2}\\ c_{3}=\beta r^{2}\\ c_{4}=0\\ \theta_{0}=K^{2}+\beta^{2}B^{2}\\ \theta_{1}-2\theta_{0}\theta_{2}=K^{2}\beta^{2}+R^{2}\\ \theta_{2}^{2}-2\theta_{1}\theta_{3}=K^{2}r^{4}+\beta^{2}B^{2}r^{4}+2\beta R^{2}\\ \theta_{3}^{2}=K^{2}\beta^{2}r^{4} \end{array}$$
Step 3 The Equation (12) can be written as a linear state space mode as follow:
(14) $$\begin{array}{cc} \dot{X}(t) & =\left[\begin{array}{cccc} 0 & 1 & 0 & 0\\ 0 & 0 & 1 & 0\\ 0 & 0 & 0 & 1\\ -c_{4} & -c_{3} & -c_{2} & -c_{1} \end{array}\right]X(t)+\left[\begin{array}{c} 0\\ 0\\ 0\\ 1 \end{array}\right]\xi (t)\\ Y(t) & =\left[\begin{array}{cccc} \theta_{3} & \theta_{2} & \theta_{1} & \theta_{0} \end{array}\right]X(t)+\upsilon (t) \end{array}$$
  where *X*(*t*) is continuous-time state vector, and the *Ẋ*(*t*) is the differential of *X*(*t*). Both ξ(*t*) and υ (*t*) are continuous-time noise, and they are white, zero-mean, uncorrelated.

The general linear state-space model for random errors of inertial sensor can be obtained by converting the Equation (14) into discrete form:

(15) $$\begin{array}{ll} X_{k} & =\Phi_{k-1}X_{k-1}+G_{k-1}\xi_{k-1}\\ Y_{k} & =\Theta_{k}X_{k}+\Psi_{k}\upsilon_{k} \end{array}$$

where *k* = 1, 2, …, *X_k_* is state vector, and *Y_k_* is the measurement. The Φ*_k_* is state-transition matrix, and Θ*_k_* is the measurement matrix. The *G_k_* and Ψ*_k_* are the process and measurement noise matrix, respectively. Here ξ*_k_* and υ*_k_* are discrete-time noise, and they are white, zero-mean, uncorrelated.

### 3.2. New Finite-Dimensional Filters

The new finite-dimensional filters were proposed by Elliott and Krishnamurthy in 1999 [18]. In a linear dynamic system, they were used with expectation maximization (EM) algorithm to yield the maximum likelihood estimation. Compared to the standard KF-EM algorithm, the new finite-dimensional filters have two main advantages. The first advantage is that the memory requirements are significantly reduced, and the second advantage is that it can be easily used in a multiprocessor system. The main process of this recursive filter is shown below, and the detailed derivations are reported in [18]. The linear state-space model that the states *x_k_* are observed indirectly via the observations *y_k_* can be written as [15,18]:

(16) $$\begin{array}{ll} x_{k} & =A_{k-1}x_{k-1}+J_{k-1}\xi_{k-1}\\ y_{k} & =C_{k}x_{k}+V_{k}\upsilon_{k} \end{array}$$

where *A_k_* is state-transition matrix, and *C_k_* is the measurement matrix. The *J_k_* and *V_k_* are the process noise and measurement noise matrix, respectively. Note that *Â_k_* and *Ĉ_k_* are the estimate of *A_k_* and *C_k_*, respectively. Both ξ*_k_* and υ*_k_* are noise that have been defined below Equation (15). The Kalman filter can be expressed as [18]:

(17) $$P_{k}^{-}={\hat{Q}}_{k-1}^{f}+{\hat{A}}_{k-1}P_{k-1}^{+}{\hat{A}}_{k-1}^{T}$$

(18) $${\hat{x}}_{k}^{+}={\hat{A}}_{k-1}{\hat{x}}_{k-1}^{+}-P_{k}^{-}{\hat{C}}_{k}^{T}{\left[{\hat{C}}_{k}P_{k}^{-}{\hat{C}}_{k}^{T}+{\hat{R}}_{k}^{f}\right]}^{-1}\left(y_{k}-C_{k}{\hat{A}}_{k-1}{\hat{x}}_{k-1}^{+}\right)$$

(19) $$P_{k}^{+}=P_{k}^{-}-P_{k}^{-}{\hat{C}}_{k}^{T}{\left[{\hat{C}}_{k}P_{k}^{-}{\hat{C}}_{k}^{T}+{\hat{R}}_{k}^{f}\right]}^{-1}{\hat{C}}_{k}P_{k}^{-}$$

where $P_{k}^{-}$ represents the *a priori* state covariance, and $P_{k}^{+}$ represents the *a posteriori* state covariance. The ${\hat{x}}_{k}^{+}$ represents the *a posteriori* state estimate, and the superscript *T* is a symbol that represents a transposed matrix. The ${\hat{Q}}_{k}^{f}$ and ${\hat{R}}_{k}^{f}$ are estimates of the process noise covariance and measurement noise covariance, respectively.

The *Z_k_*, *O_k_*, and *S_k_* are the notations used for simplifying the equation and are defined as follows:

(20) $$Z_{k}^{-1}=P_{k}^{+}-P_{k}^{+}{\hat{A}}_{k-1}^{T}{\left(P_{k}^{-}\right)}^{-1}{\hat{A}}_{k-1}P_{k}^{+}$$

(21) $$O_{k}={\left(P_{k}^{-}\right)}^{-1}{\hat{A}}_{k-1}P_{k}^{+}$$

(22) $$S_{k}=Z_{k}^{-1}{\left(P_{k}^{+}\right)}^{-1}{\hat{x}}_{k}^{+}$$

Calculate the $a_{k}^{ij(M)}$, $b_{k}^{ij(M)}$ and $d_{k}^{ij(M)}$, *M* = 0, 1, 2 as follows:

(23) $$a_{k+1}^{ij(0)}=a_{k}^{ij(0)}+b_{k}^{ij{\left(0\right)}^{T}}S_{k}+Tr\left[d_{k}^{ij(0)}Z_{k}^{-1}\right]+S_{k}^{T}d_{k}^{ij(0)}S_{k}$$

(24) $$b_{k+1}^{ij(0)}=O_{k}\left(b_{k}^{ij(0)}+2d_{k}^{ij(0)}S_{k}\right)$$

(25) $$d_{k+1}^{ij(0)}=O_{k}d_{k}^{ij(0)}O_{k}^{T}+\frac{1}{2}\left(e_{i}{e_{j}}^{T}+e_{j}{e_{i}}^{T}\right)$$

(26) $$a_{k+1}^{ij(1)}=a_{k}^{ij(1)}+b_{k}^{ij{\left(1\right)}^{T}}S_{k}+Tr\left[d_{k}^{ij(1)}Z_{k}^{-1}\right]+S_{k}^{T}d_{k}^{ij(1)}S_{k}$$

(27) $$b_{k+1}^{ij(1)}=O_{k}(b_{k}^{ij(1)}+2d_{k}^{ij(1)}S_{k})+e_{i}{e_{j}}^{T}S_{k}$$

(28) $$d_{k+1}^{ij(1)}=O_{k}d_{k}^{ij(1)}O_{k}^{T}+\frac{1}{2}\left(e_{i}{e_{j}}^{T}O_{k}^{T}+O_{k}e_{j}{e_{i}}^{T}\right)$$

(29) $$a_{k+1}^{ij(2)}=a_{k}^{ij(2)}+b_{k}^{ij{\left(2\right)}^{T}}s_{K}+Tr\left[d_{k}^{ij(2)}Z_{k}^{-1}\right]+S_{k}^{T}\left(d_{k}^{ij(2)}+e_{i}{e_{j}}^{T}\right)S_{k}+Tr\left[e_{i}{e_{j}}^{T}Z_{k}^{-1}\right]$$

(30) $$b_{k+1}^{ij(2)}=O_{k}\left(b_{k}^{ij(2)}+2\left(d_{k}^{ij(2)}+e_{j}{e_{i}}^{T}\right)S_{k}\right)$$

(31) $$d_{k+1}^{ij(2)}=O_{k}\left(d_{k}^{ij(2)}+\frac{1}{2}\left(e_{i}{e_{j}}^{T}+e_{j}{e_{i}}^{T}\right)\right)O_{k}^{T}$$

(32) $$a_{k+1}^{in}=a_{k}^{in}+b_{k}^{in^{T}}S_{k}$$

(33) $$b_{k+1}^{in}=O_{k}b_{k}^{in}+e_{i}\langle y_{k+1},e_{n}\rangle$$

where *i*, *j* ∈ {1, 2, …, *m*}, *n* ϵ {1, 2, …, *n*}, *e_i_*, *e_j_*, *e_n_* denote the unit column vectors in the *i*th, *j*th, and *n*th columns, respectively. Here Tr [] denotes the trace of matrix, and 〈·,·〉 denotes the inner product.

The initializations of the above equations are defined as follows:

(34) $$\begin{array}{ll} a_{0}^{ij(0)} & =a_{0}^{ij(1)}=a_{0}^{ij(2)}=0\\ b_{0}^{ij(0)} & =b_{0}^{ij(1)}=b_{0}^{ij(2)}=0_{m\times 1}\\ d_{0}^{ij(0)} & =\frac{e_{i}e_{j}^{T}+e_{j}e_{i}^{T}}{2}\\ d_{0}^{ij(1)} & =d_{0}^{ij(1)}=0_{m\times m}\\ a_{0}^{in} & =0\\ b_{0}^{in} & =e_{i}\langle y_{o},e_{n}\rangle \end{array}$$

The *Â_k_*, ${\hat{Q}}_{k}^{f}$, ${\hat{C}}_{k+1}$, and ${\hat{R}}_{k+1}^{f}$ can be expressed as follows:

(35) $${\hat{A}}_{k}=U_{k}^{\left(1\right)}{\left(U_{k}^{\left(2\right)}\right)}^{-1}$$

(36) $${\hat{Q}}_{k}^{f}=\frac{1}{k}\left(U_{k}^{\left(0\right)}-{\hat{A}}_{k}U_{k}^{{\left(1\right)}^{T}}-U_{k}^{\left(1\right)}{\hat{A}}_{k}^{T}-{\hat{A}}_{k}U_{T}^{\left(2\right)}{\hat{A}}_{k}^{T}\right)$$

(37) $${\hat{C}}_{k+1}=E_{k}{\left(U_{k}^{\left(0\right)}\right)}^{-1}$$

(38) $${\hat{R}}_{k+1}^{f}=\frac{1}{k+1}\left(\sum_{m=0}^{k}y_{m}y_{m}^{T}-E_{k}^{T}{\hat{C}}_{k+1}^{T}-{\hat{C}}_{k+1}E_{k}+{\hat{C}}_{k+1}U_{k}^{\left(0\right)}{\hat{C}}_{k+1}^{T}\right)$$

where $U_{k}^{ij(M)}$ and $E_{k}^{in}$ are calculated as follows:

(39) $$U_{k}^{ij(M)}=a_{k}^{ij(M)}+b_{k}^{ij{\left(M\right)}^{T}}{\hat{x}}_{k}^{+}+Tr\left[d_{k}^{ij(M)}P_{k}^{+}\right]+{\left({\hat{x}}_{k}^{+}\right)}^{T}d_{k}^{ij(M)}{\hat{x}}_{k}^{+}$$

(40) $$E_{k}^{in}=a_{k}^{in}+b_{k}^{in^{T}}{\hat{x}}_{k}^{+}$$

As shown in [15], the major drawback of this method is that the quantization noise cannot be directly incorporated into the error model because the equivalent ARMA model used here is driven with white noise [23].

## 4. New Method

As shown in Subsection 3.1, the modeling of stochastic errors in the existing method is indirect. This is because an equivalent ARMA theory was used to model the stochastic errors to obtain a linear state-space model. Moreover, the estimation process of the existing online estimation method is complex, particularly for the estimation of four parameters. In this situation, it is difficult to obtain the solution of Equation (12) and tedious to calculate Equations (23)–(33) described in Subsection 3.2. Focusing on the disadvantages and inspired by the expression of $\delta_{\text{total}}^{2}\left(\tau \right)$, which is nonlinear in nature, a simple and direct online estimation method based on a nonlinear state-space model and NEKF is proposed in the following two subsections.

### 4.1. New Nonlinear State-Space Model of Allan Variance Coefficients

To estimate the Allan variance coefficients directly in real time, $\delta_{\text{total}}^{2}(\tau )$ should be a dynamic expression at discrete-time *k*. The recursive algorithm is the best choice for online estimation because the computation can be carried out as soon as a new sample arrives. The detailed derivation of the recursive formulation for Allan variance is shown below:

Assume ℜ is the total number of data points. According to [5,6], the two steps of Allan variance computation can be expressed as follows:

(41) $${\bar{w}}_{k}(l)=\frac{1}{l}\sum_{\bar{i}=1}^{l}w_{k}(\bar{i});k=1,2,\cdots ,L$$

(42) $$\delta_{\text{total}}^{2}(\tau_{\bar{m}})=\frac{1}{2}\langle {\left({\bar{w}}_{k+1}(l)-{\bar{w}}_{k}(l)\right)}^{2}\rangle ≅\frac{1}{2(L-1)}\sum_{k=1}^{l-1}{\left({\bar{w}}_{k+1}(l)-{\bar{w}}_{k}(l)\right)}^{2}$$

where *l* is the length of the data cluster, and *L* = ℜ/*l* is the number of data clusters. Recall that, the τ is sample time that has been defined in Section 2, and *τ_m̅_* = *lτ* is the correlation time.

Equation (41) shows the average of each cluster, and each of them can be rewritten in the recursive form as follows:

(43) $$\begin{array}{ll} {\bar{w}}_{k}(\bar{m}) & =\frac{1}{\bar{m}}\overset{\bar{m}}{\underset{\bar{j}=1}{\text{∑}}}w_{k}(\bar{j})\\ & =\frac{w_{k}(\bar{m})}{\bar{m}}+\left(\frac{\bar{m}-1}{\bar{m}}\right)\left(\frac{1}{\bar{m}-1}\right)\overset{\bar{m}-1}{\underset{\bar{j}=1}{\text{∑}}}w_{k}(\bar{j})\\ & =\frac{1}{\bar{m}}w_{k}(\bar{m})+\left(\frac{\bar{m}-1}{\bar{m}}\right){\bar{w}}_{k}(\bar{m}-1) \end{array}$$

where 1 ≤ *m̅* ≤ *l*, and *w̅_k_* (0) = 0.

Equation (42) shows the Allan variance, and each of the recursion equation can be written as follows:

(44) $$\begin{array}{ll} \delta_{k}^{2}(\bar{m}) & =\frac{1}{2(k-1)}\overset{k-1}{\underset{\bar{d}=1}{\text{∑}}}{\left({\bar{w}}_{\bar{d}+1}(\bar{m})-{\bar{w}}_{\bar{d}}(\bar{m})\right)}^{2}\\ & =\frac{1}{2(k-1)}{\left({\bar{w}}_{k}(\bar{m})-{\bar{w}}_{k-1}(\bar{m})\right)}^{2}+\left(\frac{k-2}{k-1}\right)\left(\frac{1}{2(k-2)}\right)\overset{k-2}{\underset{\bar{d}=1}{\text{∑}}}{\left({\bar{w}}_{\bar{d}+1}(\bar{m})-{\bar{w}}_{\bar{d}}(\bar{m})\right)}^{2}\\ & =\left(\frac{k-2}{k-1}\right)\delta_{k-1}^{2}(\bar{m})+\frac{1}{2(k-1)}{\left({\bar{w}}_{k}(\bar{m})-{\bar{w}}_{k-1}(\bar{m})\right)}^{2} \end{array}$$

where *k* ≥ 2 and $\delta_{1}^{2}(\bar{m})=0$.

When *l* = 1, the Allan variance of each data point can be calculated using Equation (44). Assuming that the Allan variance coefficients are composed of true values and the zero-mean Gauss white noise. Therefore, they can be expressed as follows:

(45) $${\left[\begin{array}{ccccc} Q_{k} & N_{k•} & B_{k•} & K_{k} & R_{k•} \end{array}\right]}^{T}={\left[\begin{array}{ccccc} Q_{k-1} & N_{k-1} & B_{k-1} & K_{k-1} & R_{k-1} \end{array}\right]}^{T}+\varsigma_{k-1}$$

where *Q_k_*, *N_k_*, *B_k_*, *K_k_* and *R_k_* represent the values of Quantization Noise, ARW, Bias Instability, RRW and Drift Rate Ramp at discrete-time *k*, and ς*_k_* is the zero-mean Gauss white noise. Let Equation (45) as the state equation.

According to [5], the Allan variance also can be expressed in another way:

(46) $$\delta_{k}^{2}(\tau_{\bar{m}})=\frac{3Q_{k}^{2}}{\tau_{\bar{m}}^{2}}+\frac{N_{k}^{2}}{\tau_{\bar{m}}}+\frac{2\ln2B_{k}^{2}}{\pi }+\frac{K_{k}^{2}\tau_{\bar{m}}}{3}+\frac{R_{k}^{2}\tau_{\bar{m}}^{2}}{2}$$

Based on Equations (44) and (46), the Allan variance with *m̅* = *l* = 1 can be expressed as follows:

(47) $$\left(\frac{k-2}{k-1}\right)\delta_{k-1}^{2}+\frac{1}{2(k-1)}{\left({\bar{w}}_{k}-{\bar{w}}_{k-1}\right)}^{2}=\frac{3Q_{k}^{2}}{\tau }+\frac{N_{k}^{2}}{\tau }+\frac{2\ln2B_{k}^{2}}{\pi }+\frac{K_{k}^{2}\tau }{3}+\frac{R_{k}^{2}\tau^{2}}{2}$$

Equation (47) is the dynamic equation of Allan variance and can be used as the measurement equation. Thus, the new nonlinear state-space model of Allan variance proposed here was completely modeled.

### 4.2. Neural-Extended Kalman Filter

NEKF is known as an online adaptive estimation system developed by incorporating the training into the state estimate [24,25]. In fact, NEKF is an extension of the neural Kalman filter (NKF), which has been used to overcome the two major limitations related to the utilization of KF for INS/GPS integrated system: (I) Accurate stochastic model for each of the sensor errors has to be accurately predefined; (II) Prior information about the covariance values of both inertial and GPS data as well as the statistical properties of each sensor system has to be accurately known [26,27]. Although the nonlinear state-model composed of Equations (45) and (47) look like a model that a EKF algorithm could be applied, the EKF cannot directly used in this nonlinear state-model. Because Equation (47) is nonlinear with *Q*, *N*, *B*, *K* and *R*, and there is not any statistical measurement noise in it. The high-order items in Taylor expansion can be seen as virtual measurement noise to compensate linearization error, however, the time-variable statistic of this noise is still unknown. The NEKF that trains neural network online can be used in nonlinear system with random noise [28], therefore, it was used to estimate the Allan variance coefficients in this study.

In a NEKF, an extended Kalman filter (EKF) is used as an estimator and a training paradigm. As an estimator, EKF estimates the states and the input and output weights of neural networks. As a training paradigm, it is driven by the same residuals as the state estimator and ensures that the residuals are as small as possible; it approximates the difference between the prior model used in the prediction steps of the estimator and the actual model dynamics [29]. In this application, the augmented state vector of NEKF contains both the state estimates and the weights (input and output) of the neural network. NEKF can improve the performance by estimating the weights of neural network, which in turn is used to modify the state estimate predictions of the filter as the measurements are processed. Based on this point, the NEKF algorithm is presented to online estimation Allan variance coefficients is presented. The main flow of NEKF are shown below:

The nonlinear system can be modeled as [28]:

(48) $$\begin{array}{ll} x_{k} & =f\left(x_{k-1}\right)+\ell_{k-1}\\ z_{k} & =h\left(x_{k}\right)+\varsigma_{k} \end{array}$$

where *x_k_* is the state vector, *f*(*x_k_*) is state function, *z_k_* is measurement vector, and *h*(*x_k_*) is the output function. The *ℓ_k_* and ζ*_k_* represent the random noise, respectively.

Step 1 The state *x_k_* is augmented as follows:
(49) $${\bar{x}}_{k}=\left[\frac{x_{k}}{W_{k}}\right]=\left[\begin{array}{c} x_{k}\\ \eta_{k}\\ \lambda_{k} \end{array}\right]$$
  where *W_k_* is the weights of neural network. It is composed of input weights η*_k_* and output weights λ*_k_*. Note that the neural network used in this study comprises only one hidden layer. Suppose that the number of *x_k_* is *q*, the number of hidden node [30] is *p*, and the number of neural network output is *u*. Therefore, the dimension of η*_k_* is (*q* × *p*) × 1, the dimension of λ*_k_* is (*p* × *u*) × 1, and the dimension of *W_k_* is (*q* × *p* + *p* × *u*) × 1.
  The NN(*x_k_*, η*_k_*, λ*_k_*) function used in this study is:
(50) $$\text{NN}\left(x_{k},\eta_{k},\lambda_{k}\right)=\sum_{s=1}^{p}{\left(\lambda_{k}\right)}_{s}\frac{1-e^{-2\overset{q\times p}{\underset{g=1}{\text{∑}}}{\left(\eta_{k}\right)}_{g}x_{k}}}{1+e^{-2\overset{q\times p}{\underset{g=1}{\text{∑}}}{\left(\eta_{k}\right)}_{g}x_{k}}}$$
  Note that the states *x_k_* are considered the input to the neural network, whereas the weights *W_k_* are the “parameters” of function approximator [28] that can be used to model something in the noise. More informance about the NN(*x_k_*, η*_k_*, λ*_k_*) can be found in [24–29].
Step 2 The general equations of the NEKF are defined as [26]:
(51) $$F_{k}={\frac{\partial f\left(x_{k-1}\right)}{\partial x_{k-1}}|}_{x_{k-1}={{\bar{x}}_{k-1}}^{+}}$$
(52) $$P_{k}^{-}=\left(\left[\begin{array}{cc} F_{k} & 0\\ 0 & I \end{array}\right]+\left[\begin{array}{c} \frac{\partial NN\left({x_{k-1}}^{+},{{\hat{\eta }}_{k-1}}^{+},{{\hat{\lambda }}_{k-1}}^{+}\right)}{{\partial {\hat{\bar{x}}}_{k-1}}^{+}}\\ 0 \end{array}\right]\right){P_{k-1}}^{+}{(\left[\begin{array}{cc} F_{k} & 0\\ 0 & I \end{array}\right]+[\begin{array}{c} \frac{\partial NN\left({x_{k-1}}^{+},{{\hat{\eta }}_{k-1}}^{+},{{\hat{\lambda }}_{k-1}}^{+}\right)}{{\partial {\hat{\bar{x}}}_{k-1}}^{+}}\\ 0 \end{array}])}^{T}+{\bar{Q}}_{k-1}$$
(53) $${\hat{\bar{x}}}_{k}^{-}=\left[\frac{{\hat{x}}_{k}^{-}}{{\hat{W}}_{k}^{-}}\right]=\left[\begin{array}{c} f\left({{\hat{x}}_{k-1}}^{+}\right)+NN\left({{\hat{x}}_{k-1}}^{+},{{\hat{\eta }}_{k-1}}^{+},{{\hat{\lambda }}_{k-1}}^{+}\right)\\ {{\hat{W}}_{k-1}}^{+} \end{array}\right]$$
(54) $$H_{k}=\left[{\frac{\partial h\left(x_{k}\right)}{\partial x_{k}}|}_{x_{k}={{\hat{x}}_{k}}^{-}}0\right]$$
(55) $${\bar{K}}_{k}={\bar{P}}_{k}^{-}{H_{k}}^{T}{\left(H_{k}{\bar{P}}_{k}^{-}{H_{k}}^{T}+{\bar{R}}_{k}\right)}^{-1}$$
(56) $${{\hat{\bar{x}}}_{k}}^{+}=\left[\frac{{{\hat{x}}_{k}}^{+}}{{{\hat{W}}_{k}}^{+}}\right]={{\hat{\bar{x}}}_{k}}^{-}+{\bar{K}}_{k}\left(z_{k}-h\left({{\hat{x}}_{k}}^{-}\right)\right)$$
(57) $${\bar{P}}_{k}^{+}=\left(I-{\bar{K}}_{k}H_{k}\right){\bar{P}}_{k}^{-}$$
  where *F_k_* is state Jacobian, and *H_k_* is measurement Jacobian. The **0** represents zero vector, *K̅_k_*is the Kalman Gain, ${\bar{P}}_{k}^{-}$ is the *a priori* state covariance, and ${\bar{P}}_{k}^{+}$ is the *a posteriori* state covariance. The ${\hat{\bar{x}}}_{k}^{-}$ is the *a priori* state estimate, ${\hat{\bar{x}}}_{k}^{+}$ is the *a posteriori* state estimate, *R̅_k_* is the measurement noise covariance, and *Q̅_k_* is the covariance of the process noise. The *I* represents the unit matrix.

## 5. Experimental Results

To evaluate the performance of the proposed method, the static data of an ADIS16405 MEMS sensor and low-precision FOG were collected. The reason this MEMS sensor was tested in the first experiment is that this sensor exhibits ARW, RRW, and bias instability [6,15]. Moreover, the existing method is also valid to estimate ARW, RRW, and bias instability; therefore, it can be used to compare the performance of the proposed method in this experiment. FOG, as a typical inertial sensor with five basic stochastic errors, was also used to verify the effect of the proposed method. As shown in [19], the existing method is invalid to estimate quantization noise. If the proposed method can accurately estimate the Allan variance coefficients of FOG, then it can be shown that the proposed method is more suitable to estimate the Allan variance coefficients of inertial sensors than the existing method. In the first experiment, we will use the proposed, existing, and traditional Allan variance methods to estimate the stochastic errors of the ADIS16405. However, because of quantization noise considered in the second experiment, we used the proposed and traditional Allan variance methods to analyze the stochastic errors of FOG. In each experiment, the main steps of the proposed method can be implemented as follows:

Step 1 Model the nonlinear state-space model based on the main stochastic errors.
Step 2 Make a preliminary estimate of the coefficients of the main stochastic errors.
Step 3 Use NEKF to estimate the Allan variance coefficients in real time.

### 5.1. Estimation for MEMS Experimental Data

**Figure 2.**
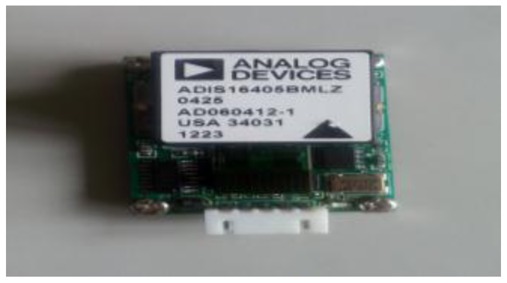
ADIS 16405.

**Figure 3.**
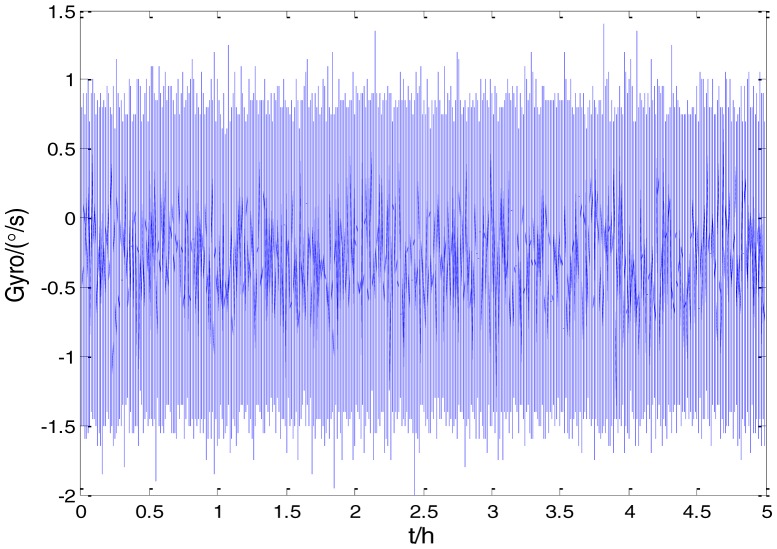
Raw data of Gyro X.

**Figure 4.**
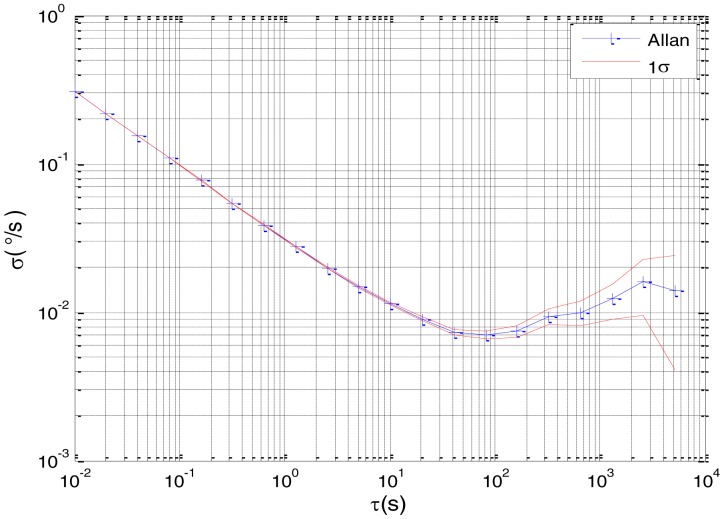
Allan variance plot of Gyro X.

**Figure 5.**
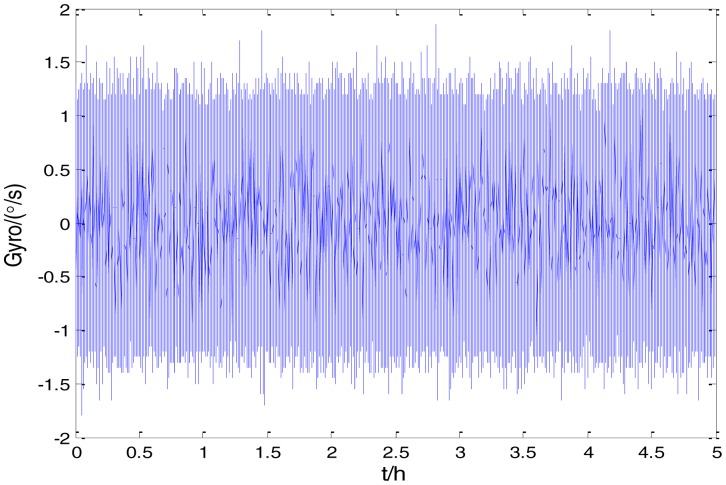
Raw data of Gyro Y.

**Figure 6.**
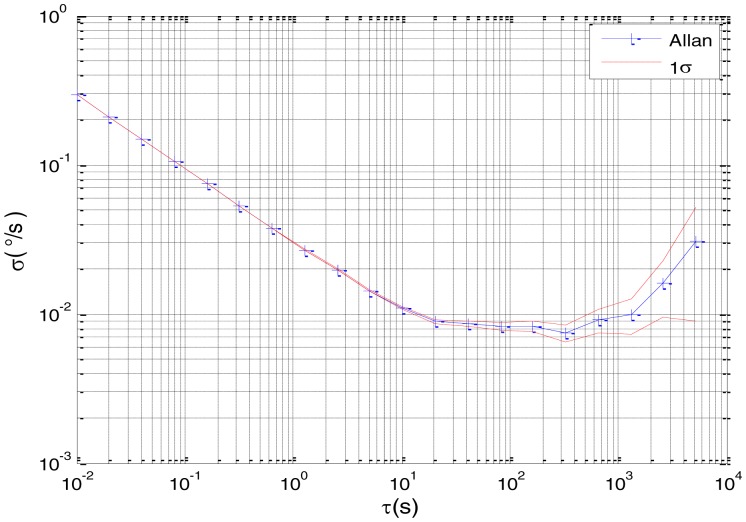
Allan variance plot of Gyro Y.

**Figure 7.**
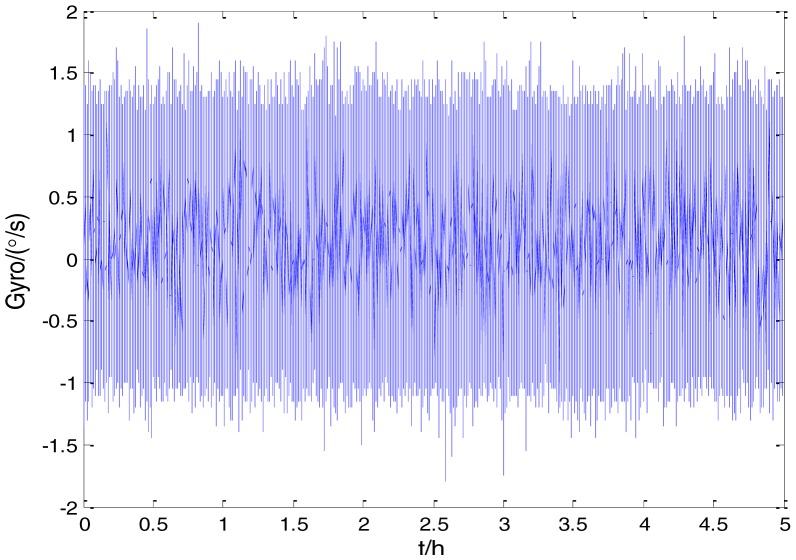
Raw data of Gyro Z.

**Figure 8.**
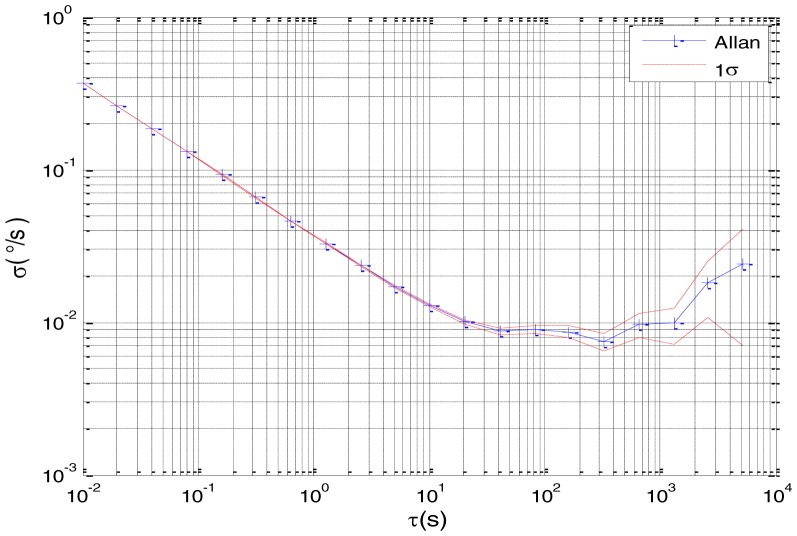
Allan variance plot of Gyro Z.

Figure 2 shows the actual MEMS sensor ADIS16405 which was used in our experiment. The 5-h static data were collected from the ADIS16405 at room temperature at 100 Hz. The raw data of gyro X, gyro Y, gyro Z and their corresponding Allan standard deviation plots are shown in Figures 3, 4, 5, 6, 7 and 8.

According to the Allan variance plot analysis, three main stochastic errors should be considered for the gyro in this test, namely, ARW, bias instability, and angular RRW with the mean-square values of *N*, *B* and *K*, respectively. Therefore, the linear state-space model of gyro can be deduced as follows:

The unified ARMA model Equation (10) consisting of ARW and bias instability can be written as follows:

(58) $$Y(t)=y_{f}(t)+y_{\text{rrw}}(t)$$

Substituting Equations (4) and (6) into Equation (58), the unified ARMA model can be written as follows:

(59) $$D(D+\beta )Y(t)=D\beta Bv_{2}(t)+(D+\beta )Kv_{3}(t)$$

According to Subsection 3.1, the Equation (59) also can be rewritten as follows:

(60) $$\ddot{Y}(t)+\sigma_{1}\dot{Y}(t)=\sigma_{2}\dot{r}+\sigma_{3}r$$

where σ_1_, σ_2_ and σ_3_ are coefficients of equivalent ARMA model. According to [15,19], the differential equation Equation (60) can be solved as follows:

(61) $$\ddot{Y}(t)+\beta \dot{Y}(t)=\sqrt{K^{2}+\beta^{2}B^{2}\dot{r}}+K\beta r$$

The corresponding state-space form of the above differential equation can be written as follows:

(62) $$\begin{array}{l} \dot{X}\left(t\right)=\left[\begin{array}{cc} 0 & 1\\ 0 & -\beta \end{array}\right]X\left(t\right)+\left[\begin{array}{c} 0\\ 1 \end{array}\right]\;\xi \left(t\right)\\ Y\left(t\right)=\left[\begin{array}{cc} K\beta & \sqrt{K^{2}+\beta^{2}B^{2}} \end{array}\right]\;X\left(t\right)+Nv\left(t\right) \end{array}$$

The discrete form of Equation (62) is:

(63) $$\begin{array}{c} X_{k}=\left[\begin{array}{cc} 1 & \tau \\ 0 & 1-\beta \tau \end{array}\right]\;X_{k-1}+\left[\begin{array}{c} 0\\ \tau \end{array}\right]\;\xi_{k-1}\\ Y_{k}=\left[\begin{array}{cc} K_{k}\beta & \sqrt{K_{k}^{2}+\beta^{2}B_{k}^{2}} \end{array}\right]\;X_{k}+\frac{N_{k}}{\tau }v_{k} \end{array}$$

where τ is the sampling interval that has been defined in Section 2. Note that Equation (63) is the linear state-space model that can be implemented by new finite-dimensional filters.

Compared to the complex process of modeling a linear state-space model, the state equation of nonlinear state-space model for Gyro can be directly written as follows:

(64) $${\bar{x}}_{k}=\left[\begin{array}{c} N_{k-1}+NN_{1}\left(x_{k-1},\eta_{k-1},\lambda_{k-1}\right)\\ B_{k-1}+NN_{2}\left(x_{k-1},\eta_{k-1},\lambda_{k-1}\right)\\ K_{k-1}+NN_{3}\left(x_{k-1},\eta_{k-1},\lambda_{k-1}\right)\\ \cdots \cdots \cdots \cdots \cdots \cdots \cdots \cdots \cdots \\ W_{k-1} \end{array}\right]+\varsigma_{k-1}$$

The first-order Taylor expansion of measurement equation Equation (47) can be written as follows:

(65) $$\left(\frac{k-2}{k-1}\right)\delta_{k-1}^{2}+\frac{1}{2(k-1)}{\left({\bar{w}}_{k}-{\bar{w}}_{k-1}\right)}^{2}=h\left({{\hat{x}}_{k}}^{-}\right)+\frac{\partial h}{\partial {{\hat{x}}_{k}}^{-}}\left(x_{k}-{{\hat{x}}_{k}}^{-}\right)+H.O.T.$$

where *H.O.T.* represents all high-order items in Taylor expansion [16], and *h*(*x_k_*); is expressed as follows:

(66) $$h\left(x_{k}\right)=\frac{N_{k}^{2}}{\tau }+\frac{2\ln2B_{k}^{2}}{\pi }+\frac{K_{k}^{2}\tau }{3}$$

To use NEKF algorithm, the Equation (65) should be written as follows:

(67) $$\left(\frac{k-2}{k-1}\right)\delta_{k-1}^{2}+\frac{1}{2(k-1)}{\left({\bar{w}}_{k}-{\bar{w}}_{k-1}\right)}^{2}=H_{k}{\bar{x}}_{k}+u_{k}+\chi_{k}$$

where:

(68) $$\begin{array}{c} H_{k}=\left[\begin{array}{cc} \frac{\partial h}{\partial {{\hat{x}}_{k}}^{-}} & 0 \end{array}\right],\left(\frac{\partial h}{\partial {{\hat{x}}_{k}}^{-}}={\frac{\partial h\left(x_{k}\right)}{\partial x_{k}}|}_{x_{k}={{\hat{x}}_{k}}^{-}}\right)\\ u_{k}=h\left({{\hat{x}}_{k}}^{-}\right)-\frac{\partial h}{\partial {{\hat{x}}_{k}}^{-}}{{\hat{x}}_{k}}^{-}\\ \chi_{k}=H.O.T. \end{array}$$

Note that χ*_k_* is the virtual measurement noise with unknown time-variable statistic to compensate linearization error H.O.T. [16].

**Figure 9.**
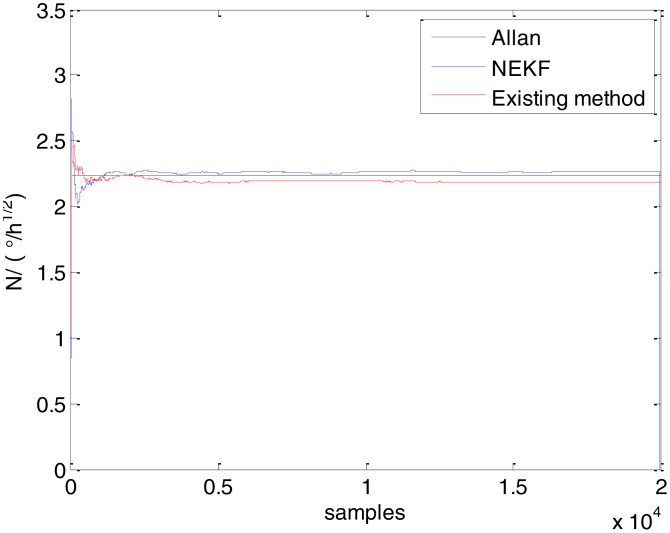
Estimates of *N* for Gyro X.

**Figure 10.**
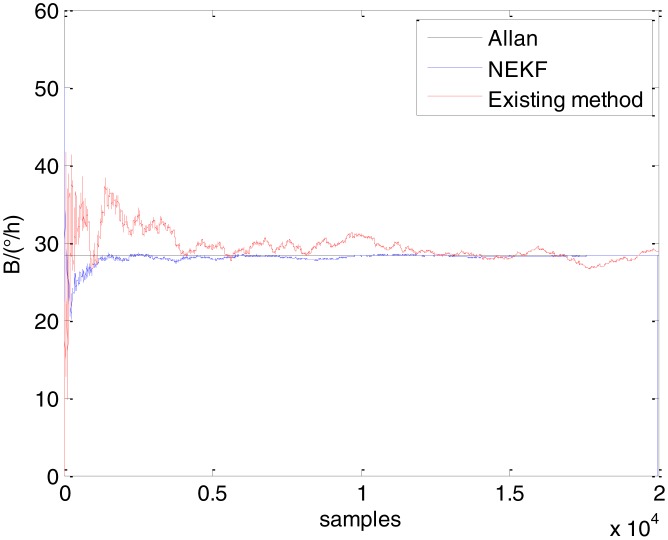
Estimates of *B* for Gyro X.

**Figure 11.**
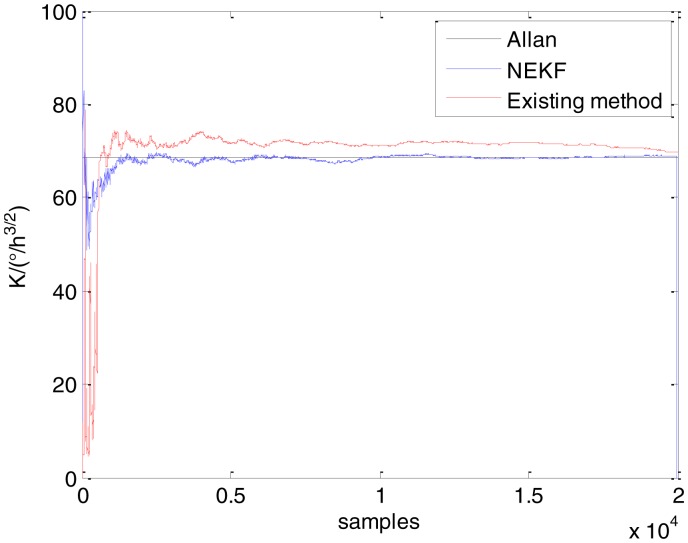
Estimates of *K* for Gyro X.

**Figure 12.**
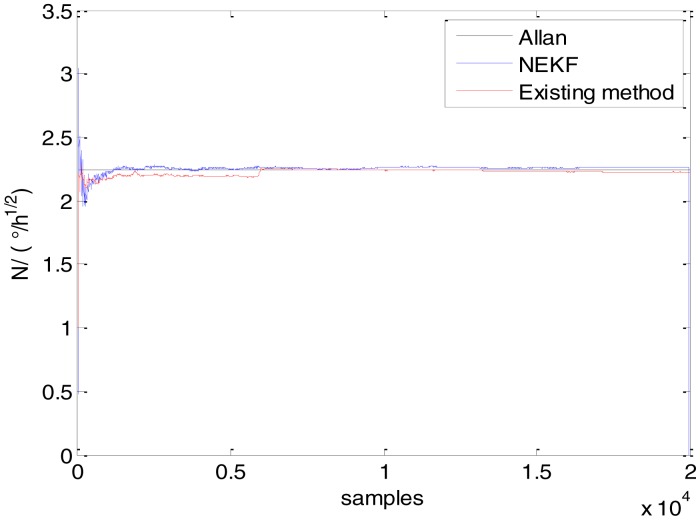
Estimates of *N* for Gyro Y.

**Figure 13.**
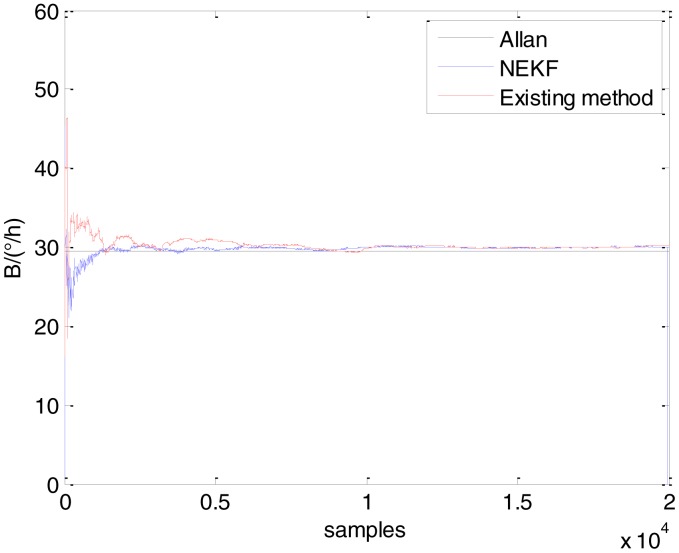
Estimates of *B* for Gyro Y.

**Figure 14.**
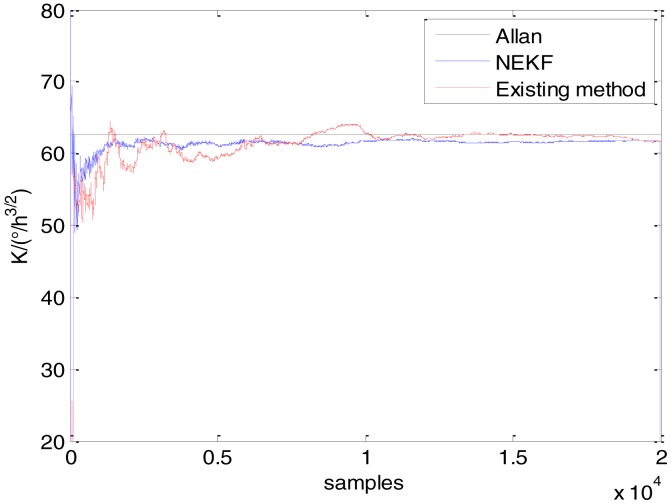
Estimates of *K* for Gyro Y.

**Figure 15.**
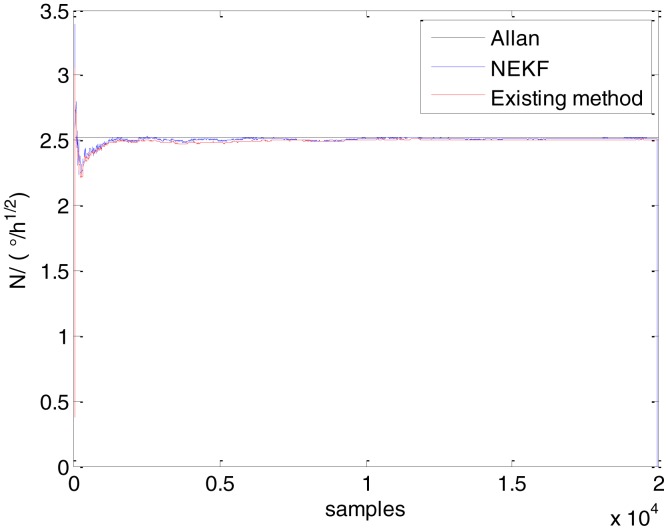
Estimates of *N* for Gyro Z.

**Figure 16.**
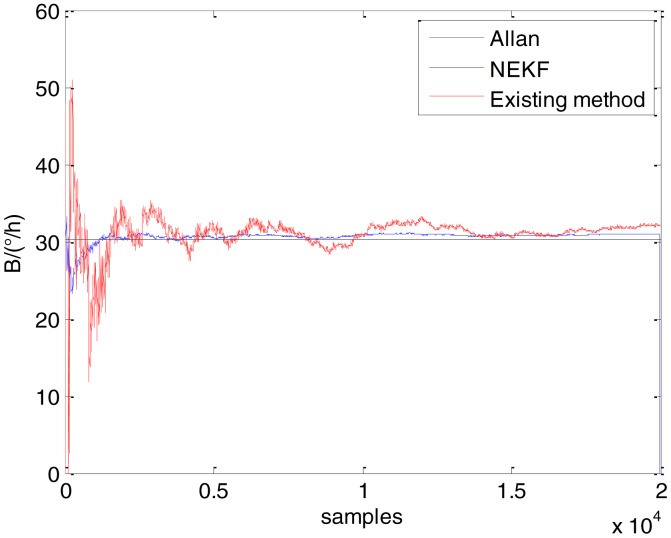
Estimates of *B* for Gyro Z.

**Figure 17.**
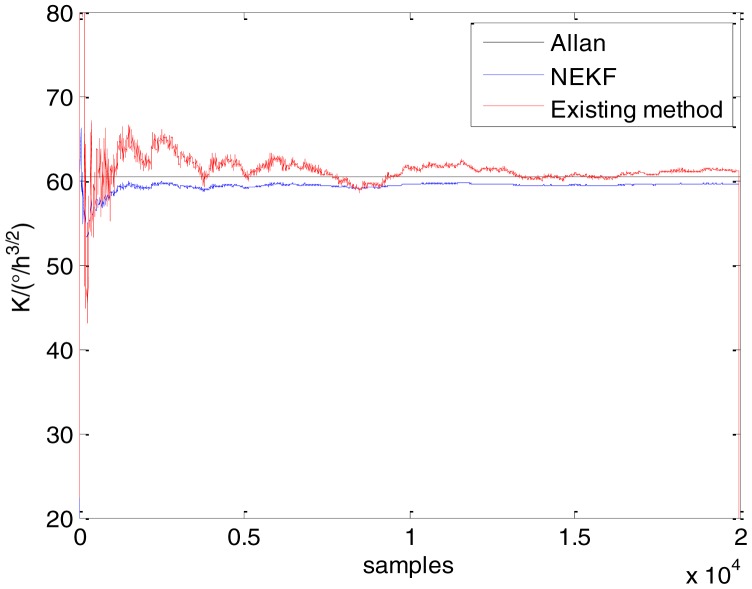
Estimates of *K* for Gyro Z.

In general, there are two methods used to set initialization. The first method is based on data sheets of inertial sensors. The second method is based on prior analysis that includes two steps: The first step is to use traditional methods to analyze the stochastic errors of sampling gyro, and the second step is to select the initial values based on the results of the first step to estimate the same type of gyros. Based on the prior analysis of ADIS 16405, the initialization of *N*, *B* and *K* were taken as 1.5 (°/*h*^1/2^), 20 (°/*h*) and 50 (°/*h*^3/2^), respectively, in this experiment. To show the initial change clearly in the estimation process, only 20,000 samples for the estimation of *N*, *B* and *K* are shown in Figures 9, 10, 11, 12, 13, 14, 15, 16 and 17.

As shown in Figures 9, 10, 11, 12, 13, 14, 15, 16 and 17, the curves of the existing method (red curve) and proposed method (blue curve) are convergent. Moreover, the convergent values of the two online estimation methods are close to those estimated by the Allan variance method. Therefore, both the existing and proposed online estimation methods can accurately estimate the coefficients of ARW, Bias Instability, and RRW in same setting conditions.

To evaluate which method (algorithm) is better, two aspects were usually considered: (1) speed and (2) accuracy. In this paper, the proposed and existing methods are online estimation methods, and they estimate the Allan variance coefficients in real time, indicating that the computation can be carried out as soon as a new sample arrives. Therefore, the speed was not compared directly here. The stability of filter used in those methods was used to evaluate which of them is better in this study. Figures 9, 10, 11, 12, 13, 14, 15, 16 and 17 show that although the trends of the two online estimation methods are basically identical, the estimation curves of existing method fluctuated significantly in the estimation process of *B* and *K*. Moreover, the standard deviation sizes of proposed method shown in Table 2 are slightly smaller than those of existing method. Therefore, the experiment suggest the NEKF in proposed method might have advantages over the new finite-dimensional filters in existing method.

As to the accuracy of the methods, Table 2 summarizes the parameter estimation results for the Allan variance, existing, and proposed methods. For a better visualization of the performance comparison, the performance indicator (|method A − method B|/method A) × 100% was defined to demonstrate the percentage of difference between methods B and A. Although the results of the Allan variance method can be affected by many factors, it is the IEEE Std [5] that is used to analyze the stochastic errors of inertial sensors. Moreover, in the online estimation of Allan variance coefficients, the results of the Allan variance method have been used as the reference values and compared to the online estimation methods proposed in [14–16]. Herein, the Allan variance method was also used as the reference method (method A), providing a baseline for comparison. According to Table 2, the performance degradation values of the proposed method over the Allan variance method are smaller than those of the existing method against the Allan variance method for *N*, *B*, and *K*, respectively.

It can also be seen that the linear state-space model was modeled using Equation (58) to Equation (63), while the nonlinear state-space model can be directly modeled in Equations (64) and (65). Therefore, in some sense, the complexity of the modeling of the proposed method is lower than that of the existing method.

### 5.2. Estimation for Fiber Optic Gyro (FOG) Experimental Data

**Figure 18.**
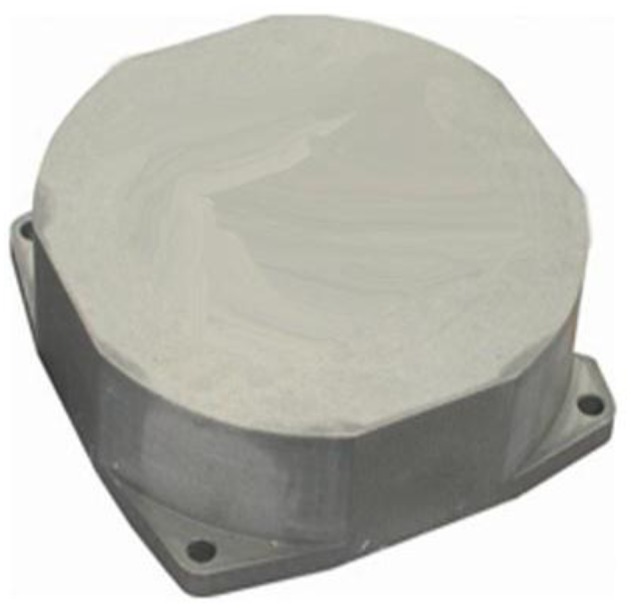
Low-precision FOG.

**Figure 19.**
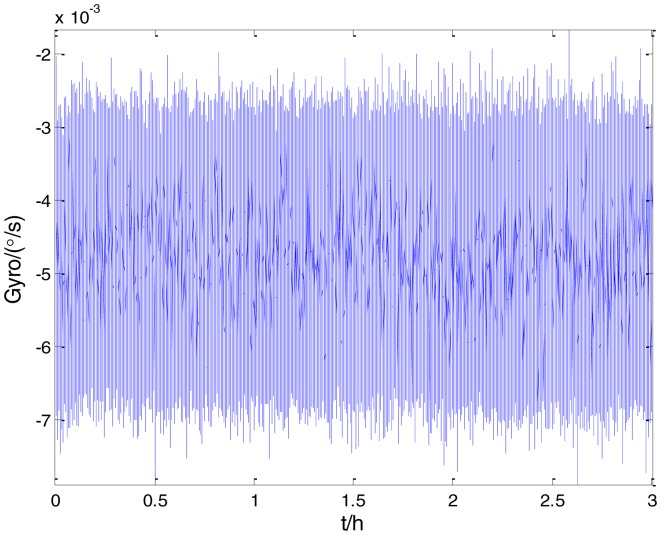
Raw data of #1 FOG.

**Figure 20.**
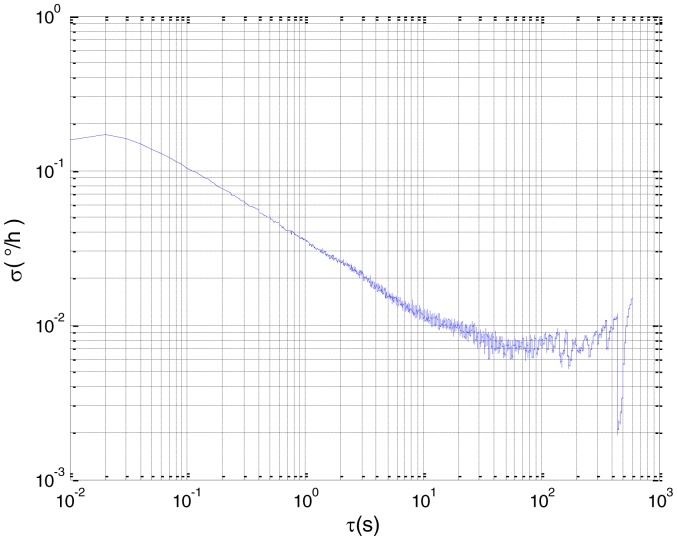
Allan variance plot of #1 FOG.

**Figure 21.**
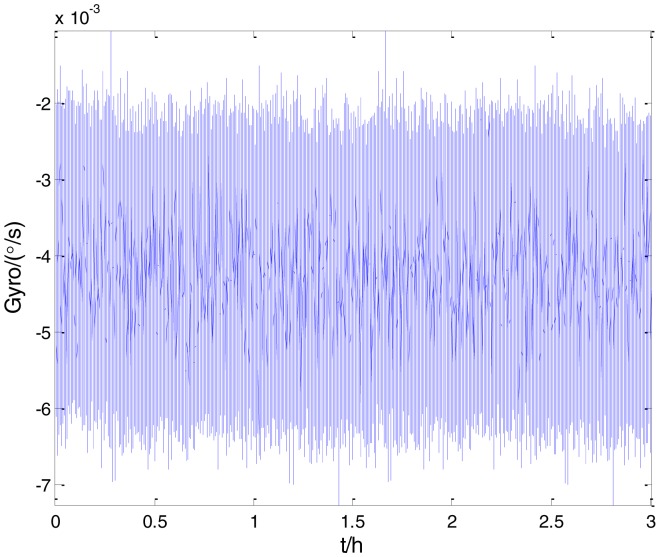
Raw data of #2 FOG.

**Figure 22.**
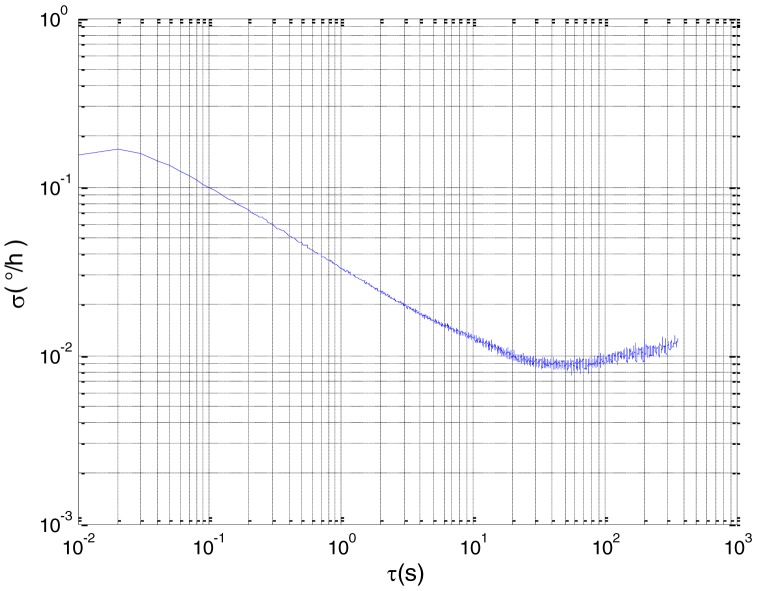
Allan variance plot of #2 FOG.

To verify that the proposed method can estimate all the five Allan variance coefficients of inertial sensors simultaneously without any limitations, the stochastic errors of the actual low-precision FOG were analyzed in this experiment. The actual low-precision FOG used in our experiment is shown in Figure 18. The 3-h static data were collected from the #1 FOG and #2 FOG at room temperature with 100 Hz. The raw data of #1 FOG and #2 FOG, and their corresponding Allan variance plots are shown in Figures 19, 20, 21 and 22.

Based on the analysis of the Allan variance plot shown in Figures 20 and 22, the five basic stochastic errors should be considered in this test. The state equation of nonlinear state-space model can be written as follows:

(69) $${\bar{x}}_{k}=\left[\begin{array}{c} Q_{k-1}+NN_{1}\left(x_{k-1},\eta_{k-1},\lambda_{k-1}\right)\\ N_{k-1}+NN_{2}\left(x_{k-1},\eta_{k-1},\lambda_{k-1}\right)\\ B_{k-1}+NN_{3}\left(x_{k-1},\eta_{k-1},\lambda_{k-1}\right)\\ K_{k-1}+NN_{4}\left(x_{k-1},\eta_{k-1},\lambda_{k-1}\right)\\ R_{k-1}+NN_{5}\left(x_{k-1},\eta_{k-1},\lambda_{k-1}\right)\\ \cdots \cdots \cdots \cdots \cdots \cdots \cdots \cdots \cdots \\ W_{k-1} \end{array}\right]+\varsigma_{k-1}$$

The measurement equation Equation (47) should be linearized by first-order Taylor expansion. The results can also be written as Equation (65). However, the *h*(*x_k_*) is:

(70) $$h\left(x_{k}\right)=\frac{3Q_{k}^{2}}{\tau^{2}}+\frac{N_{k}^{2}}{\tau }+\frac{2\ln2B_{k}^{2}}{\pi }+\frac{K_{k}^{2}\tau }{3}+\frac{R_{k}^{2}\tau^{2}}{2}$$

To use the NEKF, the first-order Taylor expansion of measurement equation Equation (47) should be written as the form of Equation (67).

**Figure 23.**
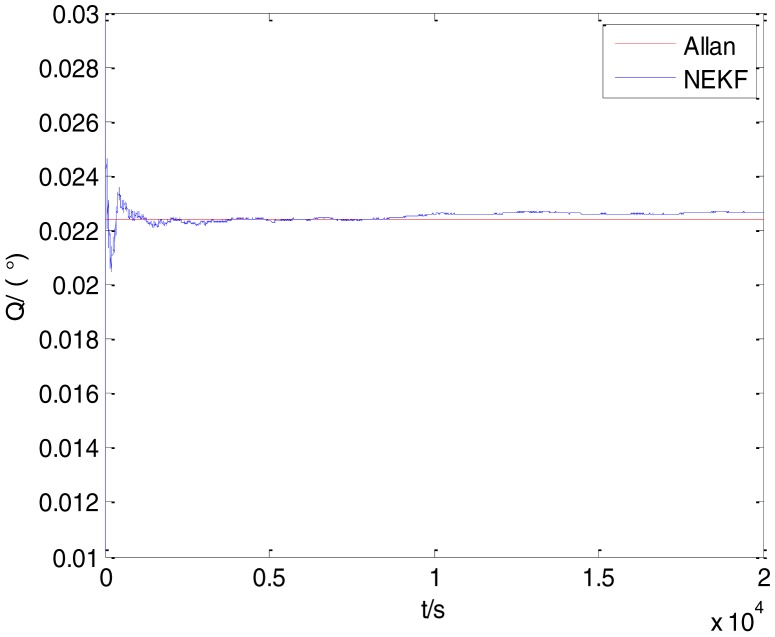
Estimation of *Q* for #1 FOG.

**Figure 24.**
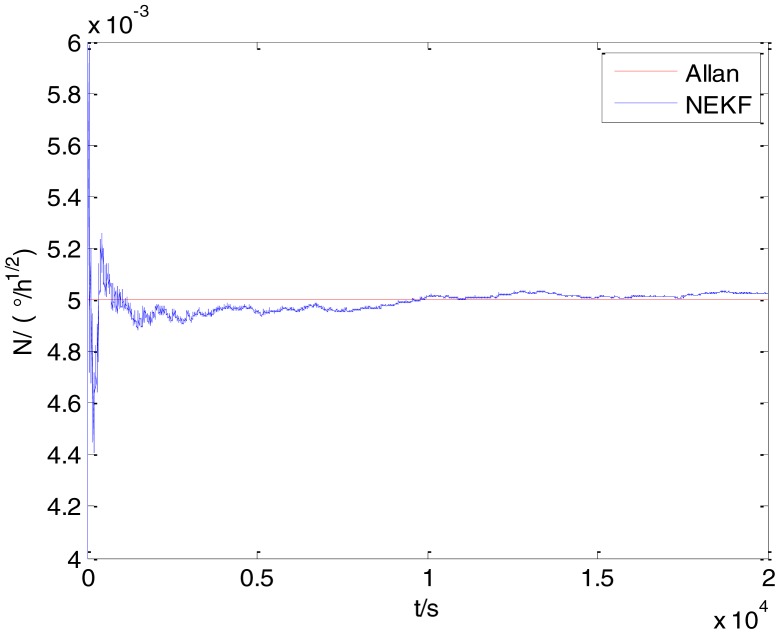
Estimation of *N* for #1 FOG.

**Figure 25.**
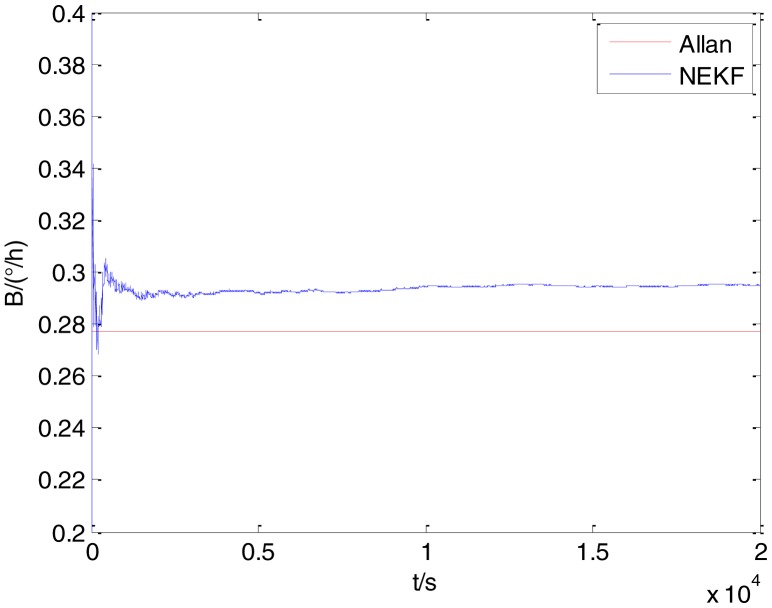
Estimation of *B* for #1 FOG.

**Figure 26.**
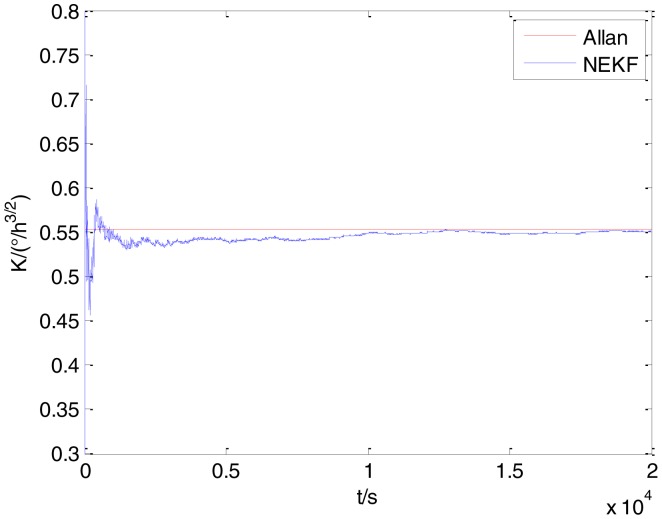
Estimation of *K* for #1 FOG.

**Figure 27.**
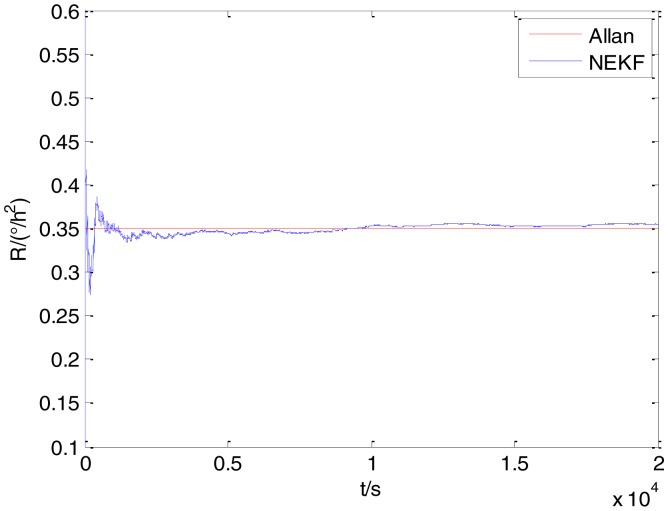
Estimation of *R* for #1 FOG.

**Figure 28.**
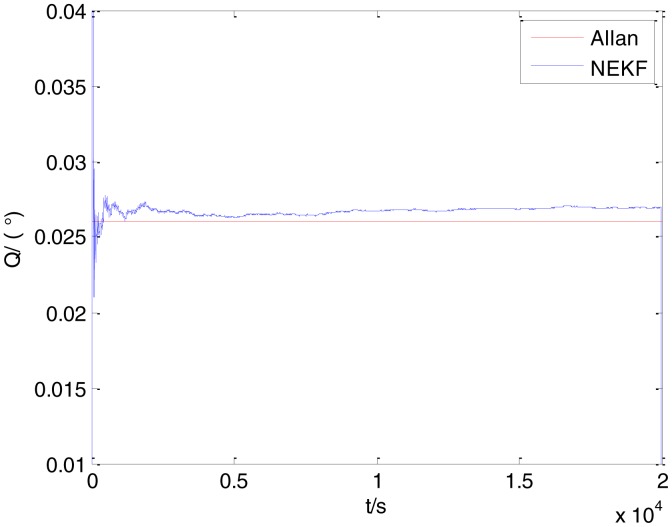
Estimation of *Q* for #2 FOG.

**Figure 29.**
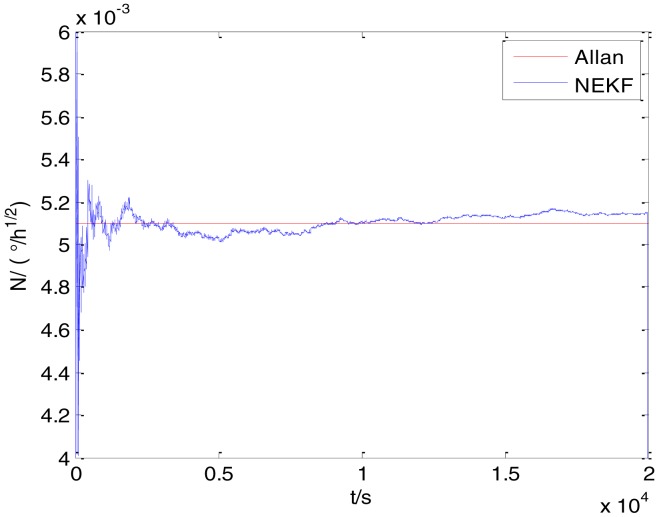
Estimation of *N* for #2 FOG.

**Figure 30.**
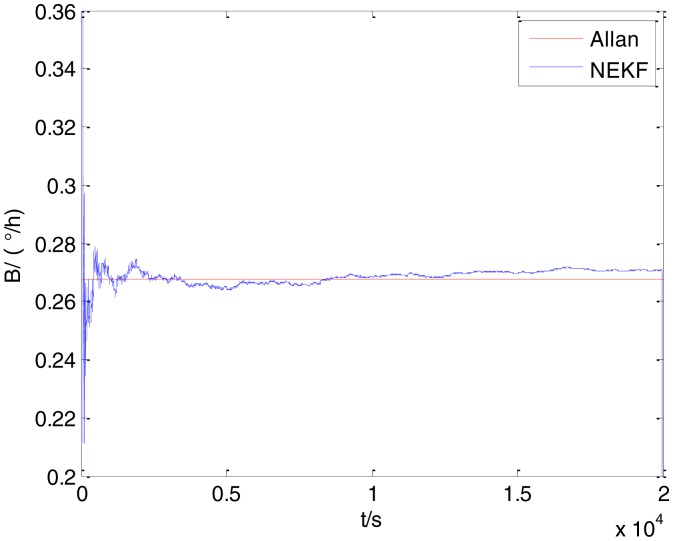
Estimation of *B* for #2 FOG.

**Figure 31.**
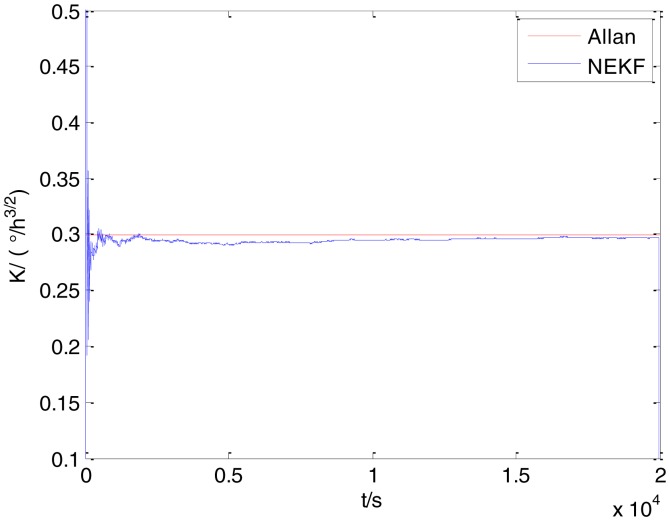
Estimation of *K* for #2 FOG.

**Figure 32.**
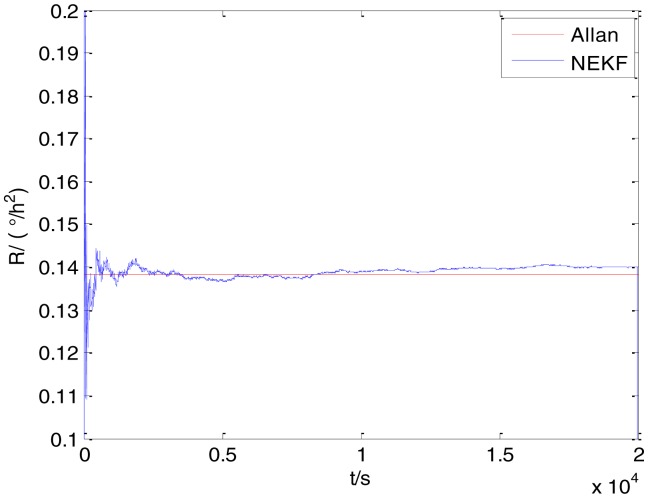
Estimation of *R* for #2 FOG.

The initialization of *Q*, *N*, *B*, *K* and *R* were taken as 0.0082(°), 0.0007(°/*h*), 0.0827(°/*h*), 0.1752(°/*h*) and 0.0547(°/*h*^2^), respectively, in this test. To show the initial change clearly in the estimation process, the simulation test curves of only 20,000 samples for the five basic stochastic errors are shown in Figures 23, 24, 25, 26, 27, 28, 29, 30, 31 and 32. The results of the classical Allan variance and the proposed methods are shown in Table 3. Figures 23, 24, 25, 26, 27, 28, 29, 30, 31 and 32 show that the estimation curves of proposed method (blue curves) are all convergent curves. Moreover, Figures 23, 24, 25, 26, 27, 28, 29, 30, 31 and 32 also show that the coefficients of five basic stochastic errors converge to the results of Allan variance method.

From Table 3, it can be seen that the mean values of five basic stochastic errors estimated by the proposed method are close to their corresponding values analyzed by Allan variance method. Therefore, this experiment prove that the proposed method can not only estimate ARW, bias instability, RRW and drift rate ramp but also valid to estimate quantization noise.

According to the above two experiments, the proposed method has a simple modeling process, and it can be used to estimate all the five basic stochastic errors. Moreover, NEKF was used in the proposed method, resulting in a better estimation results.

## 6. Conclusions and Future Work

In this paper, a new online estimation method based on a nonlinear state-space model and NEKF is proposed: the model was used instead of the traditional linear state-space model and complex finite-dimensional filter algorithm. In examination of ADIS 16405 gyro data, the proposed method performed favourably compared to the existing online method, relative to the baseline estimates obtained from the Allan variance method. In the actual FOG gyro data, the proposed method could estimate all the five basic stochastic errors simultaneously. Moreover, unlike the offline methods, the proposed method avoids the storage of a large amount of data and analyzes the Allan variance graph manually.

The success of the proposed method shows an encouraging direction in accurately estimating the Allan variance parameters for inertial sensors with recursive online analysis. However, although the proposed method performs well for static data, the onboard performance is not known. Theoretically, the new method can be used for the autonomous estimation of the Allan variance coefficients for onboard inertial sensors. However, in practice, it should be used carefully because the online estimation method can be affected by the initial values of the parameters, noise mean, and variance. The onboard performance analysis and improvement of the new online method will be studied in the future.

## Figures and Tables

**Table 1.** Summary of characteristic noise coefficients and curve slopes.

**Noise Type**	**Noise Coefficient**	**Curve Slope**
Quantization Noise	*Q*	−1
Angle Random Walk	*N*	−1/2
Bias Instability	*B*	0
Rate Random Walk	*K*	1/2
Drift Rate Ramp	*R*	1

**Table 2.** Parameter estimation results of different methods.

**Item**		**Allan Variance**	**Existing Method**			**Proposed Method**		

			**Mean**	**Indicator (%)**	**Standard Deviation**	**Mean**	**Indicator (%)**	**Standard Deviation**
Gyro X	*N_estimation_*(°/*h*^1/2^)	2.2378	2.1951	1.9081	0.0374	2.2576	0.8848	0.0362
	*B_estimation_*(°/*h*)	28.3271	30.2237	6.6954	6.6221	28.0384	1.0191	1.0077
	*K_estimation_*(°/*h*^3/2^)	68.5726	70.0132	2.1008	5.0036	68.1427	0.6269	1.7449

Gyro Y	*N_estimation_*(°/*h*^1/2^)	2.2437	2.2219	0.9716	0.0358	2.2542	0.4680	0.0400
	*B_estimation_*(°/*h*)	29.4976	30.2899	2.6860	2.1674	29.7512	0.8597	0.7754
	*K_estimation_*(°/*h*^3/2^)	62.6242	61.1463	2.3600	4.3755	61.3556	2.0257	1.5221

Gyro Z	*N_estimation_*(°/*h*^1/2^)	2.5248	2.4923	1.2872	0.0458	2.5050	0.7842	0.0398
	*B_estimation_*(°/*h*)	30.2804	31.0545	2.5564	8.3048	30.6135	1.1001	1.2304
	*K_estimation_*(°/*h*^3/2^)	60.3588	61.6976	2.2181	6.8390	59.3297	1.7050	0.9837

**Table 3.** Results of estimated parameters by the Allan variance and proposed methods.

**Item**	**#1 FOG**				**#2 FOG**			

	**Allan Variance**	**Proposed Method**			**Allan Variance**	**Proposed Method**		
	
		**Mean**	**Indicator (%)**	**Standard Deviation**		**Mean**	**Indicator (%)**	**Standard Deviation**
*Q_estimation_*(°)	0.0224	0.0226	0.8929	0.0121	0.0260	0.0267	2.6923	0.0232
*N_estimation_*(°/*h*^1/2^)	0.0050	0.0050	0.0000	0.0209	0.0051	0.0051	0.0000	0.0183
*B_estimation_*(°/*h*)	0.2771	0.2963	6.9289	0.0237	0.2667	0.2709	1.5748	0.0316
*K_estimation_*(°/*h*^3/2^)	0.5530	0.5630	1.8083	0.0305	0.2994	0.2873	4.0414	0.0220
*R_estimation_*(°/*h*^2^)	0.3499	0.3546	1.3432	0.0128	0.1382	0.1390	0.5789	0.0117
